# Epidemiology of type 2 diabetes mellitus and treatment utilization patterns among the elderly from the first wave of Longitudinal Aging study in India (2017-18)using a Heckman selection model

**DOI:** 10.1186/s12889-023-15661-4

**Published:** 2023-04-14

**Authors:** Papai Barman, Milan Das, Madhur Verma

**Affiliations:** 1 International Institute for Population Sciences (IIPS), Mumbai, India; 2 Department of community & Family medicine, All India institute of medical sciences Bathinda, Bathinda, India

**Keywords:** Diabetes mellitus, Treatment utilization, Drug adherence, Health equity, Geriatrics

## Abstract

**Introduction:**

Unmanaged Type 2 diabetes mellitus (T2DM) substantially contributes to the multi-morbidity of the elderly. Fewer research has concentrated on understanding the determinants of treatment utilization among older people, with even lesser concerns about missing data in outcome variables leading to biased estimates. The present study intends to evaluate the epidemiology of T2DM in the elderly in India and explore the socioeconomic and behavioral risk factors determining the treatment utilization among the elderly > 60 years in India by addressing the missing data to generate robust estimates.

**Methods:**

The secondary analysis used data from the Longitudinal Ageing Study in India. The key dependent variables were the presence or absence of T2DM and treatment utilization. Descriptive statistics were used to understand the differences in the prevalence of diabetes and the utilization of treatment across various socio-demographic characteristics. Heckman’s statistical technique evaluated the predictors of T2DM and treatment utilization. Analysis was done using STATA software version 14.0.

**Results:**

Almost 14% elderly reported to be living with T2DM. The odds of living with T2DM increased with non-working status, a sedentary lifestyle, and a higher BMI. A higher proportion of the elderly was on oral drugs than insulin and had been practicing lifestyle modifications to control their disease. The probability of developing T2DM was lower among females than males, but females had better odds for treatment utilization of health medication than males. Lastly, treatment utilization was significantly affected by socio-demographic characteristics like education and monthly per capita expenditure.

**Conclusions:**

Treatment utilization by the elderly living with T2DM is significantly affected by socio-demographic characteristics. Keeping in mind the increasing proportion of the geriatric population in our country, it is pertinent to tailor-made counseling sessions for the elderly to improve medication utilization and adherence and realize our goals concerning non-communicable diseases.

## Introduction

Diabetes mellitus is a debilitating, chronic condition that develops when the pancreas does not produce enough insulin or the body cannot effectively utilize it [1]. Within the last few years, diabetes mellitus has been designated one of the pressing public health priorities worldwide, including in India. The burden of Type 2 Diabetes Mellitus (T2DM) in India is massive, and with 77 million people, the country ranks second for having the highest number of T2DM patients in the world, following China. Arokiasamy et al. (2018) argued that the incidence of NCDs, particularly diabetes, was underestimated in India due to the survey’s data quality. A forecasting analysis suggests that the estimated burden figure will increase to 134 million by 2045 [2]. Overall, the prevalence of diabetes varies significantly by age group, with older people at greater risk than younger people [3]. With the advent of COVID-19, the increased mortality in people living with T2DM has further raised concerns about disease control, especially in the elderly.

The Sustainable Development Goals (MDGs) envisage reducing the percentage of premature deaths caused by NCDs, including diabetes, to one-third by 2030 [4]. On the same lines, India’s national health policy (2017) intends to enhance the screening and treatment of those with diabetes by 80% and 25% by 2025 [5]. The economic burden of T2DM is overwhelming for patients and the health systems and is seen as a significant bottleneck in realizing our diabetes-related goals. This is attributed to the rising prevalence of people living with T2DM due to the chronic nature of the disease and increased complications. Also, there has been a drastic change in how different anti-diabetic classes have been used recently, due to which the cost of medical care due to T2DM has escalated in India [6]. Also, certain social factors that affect health outcomes, such as low income, job insecurity, low educational attainment, and poor living conditions, significantly impact diabetes [7].

Disease management has two components. One is the availability of treatment, and the other is taking up the treatment. Fewer research has concentrated on understanding the determinants of treatment utilization among older people living with T2DM in India. The decision to use medicine and seek care related to T2DM is correlated with many other known and unknown factors. A previous study from India collected information regarding the barriers to optimum treatment utilization by people living with diabetes in India but was limited to insulin usage. As per the study, the greatest barrier to insulin therapy from the patient’s perspective was pain and fear of using injectable modality; treatment cost; loss to follow-up; lack of adequate counseling; and treatment compliance [8]. Most information about these factors is collected through cross-sectional observational studies conducted at national and sub-national levels. However, a growing body of scientific evidence has emphasized the importance of potential confounders in such research studies. The use of naïve models may produce biased underestimates of such confounding factors. Further, many medical researchers are less acquainted with the concepts of missing data and techniques to address them. Therefore, a considerable degree of missing data in outcome variables is often neglected while making scientific inferences [9]. This missing data in the variable of interest is critical in clinical data in low-income settings, where accurate measures of clinical outcomes are often only available for a relatively small proportion of the population [10]. Even though missing values can be imputed using multiple imputations, this approach can lead to biased estimates if confounding factors affect both the outcome of interest and the likelihood of missing data [11].

Within this context, data from the Longitudinal Ageing Study in India, 2017, allows us to assess the frequency of treatment utilization by the people living with T2DM in India. Thus the present study intends to evaluate the prevalence of T2DM in the elderly in India and explore the socioeconomic and behavioral risk factors determining the medication uptake among persons aged 60 and over in India by addressing the missing data to give robust estimates.

## Methodology

### Data source

The present study utilized secondary data from the Longitudinal Ageing Study in India (LASI, Wave-1, 2017-18), which used a multi-stage stratified probability sampling design in which three stages for rural areas and four stages for urban areas and covered the whole of India [12]. In the first stage, it chose a primary sample unit (PSU); in the second, a village for rural areas and a ward for urban areas. In the third, a household was selected from each chosen village and a census enumeration block (CEB) was selected from the chosen ward, and in the fourth, a household was selected from each chosen CEB. The fourth stage was utilized for only urban areas. When it finally sampled for individuals, the actual response rate was 87.3. All other procedures, including the sampling framework and more details about selecting household and individual, is available in the LASI report [13]. It collected immense information on demographic, social, and economic conditions, chronic and mental health, and healthcare utilization from individuals.

### Study participants

The survey included 72,250 individuals aged 45 years and above and their spouses from each household, and among them 31,902 older persons were aged 60 years and above. In the original survey, a person who has lived at the place of enumeration (this house) for six months or more during the last year or intends to live there for at least six months in the next year was included. This included a pregnant woman who has gone to her natal house for delivery and is expected to return to the household soon and ‘rotating’ parents or older people who have lived there for more than six months during the last year. On the other hand, the person who has neither lived at the place of enumeration for six months during the last year nor intends to live there for at least six months in the future, and people living in institutions such as old age homes, mental asylums, ashrams, and religious homes, and incarcerated people (prison inmates) were excluded in the LASI sampling realm.

In the present study, since the objectives concerned the elderly, we only included participants above 60 years of age, while the older adults (45–60 years) who participated in the survey were excluded. Therefore, around 27,386 samples were included in the final analysis after excluding the missing cases.

### Study variables

The key dependent variables were the presence or absence of T2DM and treatment utilization. In LASI, the variables were derived by asking the respondent whether “he/she was ever diagnosed with diabetes or high blood sugar,” The answer was coded as 0 for ‘no’ and 1 for ‘yes.’ It also asked three questions: “a. to treat or control your diabetes or high blood sugar, are you currently taking medications that you swallow?”; “b. are you currently using insulin shots/injections?”; and “are you following a special diet? All the possible answers were collected in binary form, 0 for ‘no’ and 1 for ‘yes.’ A single variable was generated using all three question’s answers as treatment in any form (no = 0 if one reported no to all three questions, otherwise, yes = 1).

Independent variables, including Demographic, and socioeconomic characteristics, were included following a literature review. We had Age (categorized in 60–69, 70–79, 80 & above age groups), sex (female and male), marital status (union included currently married and living in relationship, not in a partnership), education level (no education, less than primary, primarily completed, secondary completed, and Higher Secondary and above), social category (scheduled caste (SC), scheduled tribe (ST), other backward class (OBC), and Others), religious type (Hindu, Muslim, and others), monthly per capita expenditure (MPCE) (poorest, poorer, middle, richer, and richest), place of residence (rural, and urban), and currently working status (no, and yes). Further, five health behavior indicators considered to be risk factors of chronic diseases were included as current smoking status (no and yes), drinking alcohol (not frequently, and frequently), engaging in vigorous activity (every day to never), moderate energetic activity(every day to never), and yoga/meditation/asana/pranayama/similar (every day to never) [14, 15], body mass index (BMI), which is considered to be associated with health risks, was also included to examine diabetes [16]. BMI was constructed based on the ratio of the weight (kg) and the square of height (meter) and categorized based on the recommendation of the World Health Organization (WHO) as underweight (< 18.5 = 1) normal (18.5–24.9 = 2), overweight (25-29.9 = 3), and obese (> 30).

### Statistical analysis

Descriptive statistics were used to understand the differences in the prevalence of diabetes and the utilization of treatment across different strata of various socio-demographic characteristics. Predictors for various types of T2DM were explored using the Heckman sample selection model [11]. Basically, the usual standard probit model includes all the individuals who reported yes to T2DM but lagged to have the individuals who said ‘no’ for diabetes. Heckman’s statistical technique holds the merit of understanding the determinants of not only the reported observations but also considers unreported observations, i.e., utilization of treatment in both scenarios, i.e., having T2DM diabetes and not. In simple language, the technique helps you to predict the determinants of treatment utilization even among those participants who had not reported T2DM in the present survey. Only the variables significant in unadjusted analysis (p < 0.1) were considered to build the final model. A variance inflation factor (VIF) was generated to check the multicollinearity among the exposure-independent variables, and no evidence of multicollinearity was found in the variables used. We accounted for the cluster-sampling design of the LASI to obtain all weighted prevalence and associated factors. All the analyses were conducted using individual weights in STATA software version 14.0.

## Results

Table 1 depicts the sample distribution of the study population. A significant proportion of the study sample was between 60 and 69 years (61.4%), female (51.7%), living in-union (64.7%), un-educated (53.6%), belonging to OBC (39.6%) caste, and following Hinduism (74.2%), living in rural areas (67.3%), and was currently not working (69.5%). Considering health behavior indicators, a substantial percentage were either currently smoking (30.9%) or frequently consuming alcohol (30.5%). While around two-thirds (68.3%) and fifth-sixth (84.6%) of the older persons were found to be not engaged in vigorous and yoga/meditation/asana/pranayama activities, respectively, around two-thirds (62.6%) of respondents were found to be involved in doing moderate activities. Nearly one-fourth (24.6%) of participants were either overweight or obese.

**Table 1:** Sample distribution among the older persons who participated in the first wave of the Longitudinal Aging Study in India (2017-18) were included in the analysis

Characteristics	Unweighted counts	Weighted Percentage (%)
**Total**	27,386	100.0
**Age (completed years)**		
60–69	16,810	61.4
70–79	7900	28.9
80 and+	2676	9.8
**Sex**		
Male	13,241	48.4
Female	14,145	51.7
**Marital Status**		
In Union	17,711	64.7
Not in Union	9675	35.3
**Educational Level**		
No Schooling	14,689	53.6
Less than Primary	3379	12.3
Less than secondary	5281	19.3
Utpo Secondary	2034	7.4
Higher secondary and Above	2003	7.3
**Social Category**		
Scheduled Caste	4573	16.7
Scheduled Tribe	4751	17.4
Other Backward Class	10,834	39.6
Other caste	7228	26.4
**Religion**		
Hindu	20,332	74.2
Muslim	2897	10.6
Other	4157	15.2
**Wealth quintile**		
Poorest	5670	20.7
Poorer	5661	20.7
Middle	5640	20.6
Richer	5394	19.7
Richest	5021	18.3
**Place of residence**		
Rural	18,433	67.3
Urban	8953	32.7
**Currently Working**		
No	19,030	69.5
Yes	8356	30.5
**Currently smoking**		
No	18,929	69.1
Yes	8457	30.9
**Currently Drinking alcohol**		
Not frequently	19,030	69.5
Frequently	8356	30.5
**Engaged in Vigorous activity**		
Every Day	5019	18.3
**Several days in Week**	1555	5.7
Monthly	2110	7.7
Never	18,702	68.3
**Engaged in Moderate energetic activity**		
Every Day	13,226	48.3
Several in Week	1897	6.9
Monthly	2030	7.4
Never	10,233	37.4
**Engaged in Yoga/ meditation/ asana/** **pranayama**		
Every Day	3050	11.1
Several in Week	442	1.6
Monthly	735	2.7
Never	23,159	84.6
**Body mass index**		
Underweight	6460	23.6
Normal	14,196	51.8
Over weight	5119	18.7
Obese	1611	5.9

Around 14.3% of the elderly were living with T2DM. Table 2 presents the prevalence of T2DM by demographic and socio-economic characteristics. The majority (15.3%) of participants who were reported to be living with T2DM were between 70 and 79 years, followed by 60–69 (14.6%) and ≥ 80 years age group (9.6%). The prevalence was reported to be higher among males (14.3%) and those who were in union (14.8%), with more years of education, i.e., secondary and above (30.2% and 29.4%). A substantially higher prevalence of T2DM was observed in OBC, other social classes, and those following religions other than Hinduism or Islam. Further, the participants from the richest MPCE (22.9%), urban areas (26.6%), and currently not working (16.8%) reported higher prevalence. The prevalence was higher among the participants who were not engaged in vigorous activity (15.5%) and moderate energetic activity (15.0%); a lower prevalence was found among current smokers and alcohol users. The prevalence was significantly higher in overweight (28.2%) and obese elderly (33.7%).

**Table 2:** Weighted prevalence of T2DM among older persons in India who participated in the first wave of the Longitudinal Aging Study in India (2017-18)

Characteristics	Unweighted Count	Weighted prevalence (%)	P-value
**Overall prevalence**	27,386	**4163 (14.3)**	
**Age**			
60–69	16,810	14.6	< 0.001
70–79	7900	15.3	
80 and+	2676	9.6	
**Sex**			
Male	13,241	14.4	< 0.01
Female	14,145	14.2	
**Marital Status**			
Union	17,711	14.8	< 0.001
Not Union	9675	13.4	
**Educational Level**			
No Schooling	14,689	8.3	< 0.001
Less than Primary	3379	15.3	
Less than secondary	5281	20.4	
Utpo Secondary	2034	30.2	
Higher secondary and Above	2003	29.4	
**Social Category**			
Scheduled Caste	4573	9.5	< 0.001
Scheduled Tribe	4751	5.5	
Other Backward Class	10,834	16.3	
Other caste	7228	17.1	
**Religion**			
Hindu	20,332	14.1	< 0.001
Mushlim	2897	13.5	
Other	4157	17.7	
**Wealth Quintile**			
Poorest	5670	10.0	< 0.001
Poorer	5661	10.4	
Middle	5640	12.8	
Richer	5394	17.6	
Richest	5021	22.9	
**Place of residence**			
Rural	18,433	9.4	< 0.001
Urban	8953	26.6	
**Currently Working**			
No	19,030	16.8	< 0.001
Yes	8356	8.9	
**Currently smoking**			
No	18,929	17.1	< 0.001
Yes	8457	8.5	
**Drinking alcohol**			
Not frequently	25,625	14.6	< 0.001
Frequently	1761	8.0	
**Engaged in Vigorous activity**			
Every Day	5019	12.7	< 0.001
**Several days in Week**	1555	9.1	
Monthly	2110	10.9	
Never	18,702	15.5	
**Engaged in Moderate energetic activity**			
Every Day	13,226	14.2	< 0.001
Several in Week	1897	11.7	
Monthly	2030	13.5	
Never	10,233	15.0	
**Engaged in Yoga/ meditation/ asana/** **pranayama**			
Every Day	3050	19.4	< 0.001
Several in Week	442	15.0	
Monthly	735	20.8	
Never	23,159	13.5	
**Body mass index**			
Underweight	6460	4.2	< 0.001
Normal	14,196	12.9	
Over weight	5119	28.3	
Obese	1611	33.7	

Table 3 presents the pattern of T2DM management among those who were living with T2DM stratified as per their socio-demographic characteristics. Most of the elderly were taking oral anti-hyperglycemic (OHA) drugs (81.8%), while one-fifth (19.6%) were using injectable insulin substitutes. Overall, three-fourths (75.9%) were using lifestyle modifications as well. OHA usage was higher in the age group 70–79 years (83.3%), females (85.3%), not-in-union (83.2%), with more years of education, belonging to social castes other than SC or ST, non-Hindu/Muslim religion (84.5%), highest MPCE quintile (86.8%), residing in urban areas (86.6%). The usage of insulin and lifestyle modifications depicted a similar pattern across all variables, except that insulin usage was higher in people following Hinduism. In contrast, lifestyle modification was higher in Muslim participants.

**Table 3:** Patterns of treatment utilization to deal with diabetes among older persons in India (n = 4162) who participated in the first wave of the Longitudinal Aging Study in India (2017-18)

Characteristics	Oral anti-hyperglycemics	Injectables/ insulin shots	Lifestyle modifications
**Total**	81.8	19.6	75.9
**Age (Completed years)_**			
60–69	81.8	19.7	76.5
70–79	83.3	21.8	77.3
80 and+	74.4	8.4	65.3
**Sex**			
Male	77.9	14.6	74.5
Female	85.3	24.2	77.3
**Marital Status**			
In Union	81.0	15.7	75.1
Not in Union	83.2	26.9	77.6
**Educational Level**			
No Schooling	77.1	10.8	67.8
Less than Primary	77.0	10.6	75.4
Less than secondary	88.0	26.4	81.0
Utpo Secondary	88.1	35.4	81.7
Higher secondary and Above	78.7	19.2	80.0
**Social Category**			
Scheduled Caste	76.4	9.6	68.1
Scheduled Tribe	74.6	9.6	66.7
Other Backward Class	84.1	25.4	79.0
Other caste	80.8	14.9	74.9
**Religion**			
Hindu	81.6	20.5	75.7
Muslim	80.9	19.2	78.9
Other	84.5	10.9	75.5
**Wealth Quintile**			
Poorest	76.5	11.2	73.1
Poorer	77.9	10.0	67.9
Middle	76.7	14.7	74.4
Richer	86.2	24.9	80.2
Richest	86.8	28.9	79.8
**Place of residence**			
Rural	76.4	11.4	69.6
Urban	86.6	27.1	81.7
**Currently Working**			
No	84.2	20.7	76.8
Yes	72.0	15.1	72.5

The Heckman sample selection model was applied to explore further the determinants of T2DM and treatment utilization after addressing the censored and uncensored observations (Table 4). Overall the model depicted satisfactory fit (Wald chi (729.23) and chi test (0.00)). In addition, the rho value was 0.82, which was more significant than zero depicting a positive correlation between the two models or between diabetes and treatment utilization. The odds of living with T2DM were higher between 70 and 79 years, male gender, in a union, with more years of education, belonging to OBC or other social castes, Muslim, or other non-Hindu religion, richest quintiles, urban areas, currently not working, non-smoking, not frequently drinking, less vigorous activities, doing less energetic activities, not doing Yoga/meditation/asana/pranayama, and being overweight or obese. Similarly, the odds of utilizing the treatment against diabetes increased with higher age, female gender, being in a union, with more years of education, Muslim religion, and belonging to the middle quintile.

**Table 4:** Determinants of treatment utilization for diabetes among older persons in India from the first wave of the Longitudinal Aging Study in India (2017-18) as per Heckman sample selection model estimates

Characteristics	Outcome Equation for Diabetes (coef.)	Selection Equation for treatment utilization (coef.)
Age		
60–69 ®	1	1
70–79	0.05** (0.99 − 0.09)	-0.05 (-0.12-0.06)*
80 and+	-0.14 ***(-0.22–0.06)	0.12 (-0.08-0.32)
**Sex**		
Male ®	1	1
Female	-0.08*** (-0.13–0.04)	0.25 (0.13–0.38)***
**Marital Status**		
Union ®	1	1
Not Union	-0.07 ***(-0.12–0.03)	-0.15 (-0.27–0.02)**
**Educational Level**		
No Schooling®	1	1
Less than Primary	0.23*** (0.16–0.29)	0.02 (-0.14-0.18)*
Less than secondary	0.26***(0.21–0.32)	0.14 (-0.02-0.29)*
Utpo Secondary	0.39***(0.31–0.46)	0.27 (0.06–0.49)**
Higher secondary and Above	0.34***(0.26–0.42)	0.23 (0.01–0.45)**
**Social Category**		
Scheduled Caste ®	1	1
Scheduled Tribe	-0.18**(-0.26–0.1)	0.04 (-0.15-0.24)
Other Backward Class	0.08**(0.02–0.14)	0.05 (-0.1-0.21)
Other caste	0.02 (-0.05-0.08)	-0.03 (-0.19-0.13)
**Religion**		
Hindu ®	1	1
Muslim	0.11***(0.05–0.18)	0.15 (-0.03-0.33)*
Other	0.04 (-0.03-0.1)	0.01 (-0.13-0.15)
**Wealth quintile**		
Poorest®	1	1
Poorer	0.06*(-0.01-0.12)	-0.01 (-0.17-0.15)*
Middle	0.13***(0.07–0.2)	0.08 (-0.09-0.25)*
Richer	0.19***(0.12–0.25)	0.05 (-0.12-0.22)
Richest	0.27***(0.21–0.34)	0.04 (-0.14-0.21)
**Place of residence**		
Rural®	1	1
Urban	0.33***(0.29–0.37)	0.1 (-0.04-0.24)
**Currently Working**		
No®	1	1
Yes	-0.30***(-0.35–0.25)	0.03 (-0.12-0.18)
**Currently smoking**		
No®	1	-
Yes	-0.18***(-0.23–0.13)	-
**Currently Drinking alcohol**		
Not frequently®	1	-
Frequently	-0.12 (-0.22–0.03)***	-
**Engaged in Vigorous activity**		
Every Day®	1	-
Several in Week	-0.06 (-0.13-0.05)	-
Monthly	-0.01 (-0.1-0.08)	-
Never	0.05 (-0.06-0.1)*	-
**Engaged in Moderate energetic activity**		
Every Day®	1	-
Several in Week	0.01 (-0.07-0.09)	-
Monthly	0.01 (-0.07-0.09)	-
Never	0.08 (0.04–0.13)***	-
**Engaged in Yoga/ meditation/** **asana/pranayama**		
Every Day®	1	-
Several in Week	0.01 (-0.14-0.16)	-
Monthly	-0.04 (-0.17-0.08)	-
Never	0.01 (-0.05-0.07)	-
**Body mass index**		
Underweight®	1	-
Normal	0.45 (0.39–0.51)***	-
Over weight	0.76 (0.69–0.83)***	-
Obese	0.80 (0.71–0.89)***	-
Constant	-1.83 (-1.95–1.71)***	1.76 (1.41–2.11)***

*Note: ®: Reference category;*

*** p < 0.01, ** p < 0.05, * p < 0.1

*Number of observations (n = 27,287), Censored observations (n = 23,131), Uncensored observations (n = 4156), Wald chi2(21): 28.05, Prob > chi2: 0.00, and Rho: 0.82*

### Discussion

We understand that cross-sectional studies are limited by their inability to explain the causation and temporality and the non-random nature of the selection bias. We applied a better statistical technique to explore the predictors of treatment utilization among the elderly living with T2DM who participated in a nationally representative survey of India and addressed the inherent selection bias to an extent. Our study details specific findings that may be useful in shaping our future policies. First, almost one in seven respondents reported living with T2DM. Second, the odds of living with T2DM increased with non-working status, a sedentary lifestyle, and a higher BMI. Third, a higher proportion of the elderly preferred OHAs over insulin and had been practicing lifestyle modifications to control their disease. The probability of developing T2DM was lower among females than males, but females had better odds for treatment utilization of health medication than males. Lastly, treatment utilization was significantly affected by socio-demographic characteristics like education and MPCE.

**Aging** in India is happening at a super-quick pace. While the doubling time was around 110 and 80 years for some European countries, it is projected to take only 20 years in India to double. In percentage terms, India’s elderly population is estimated to rise from 8.6% in the 2011 census to 13.1% by 2030 [17, 18]. Therefore, a T2DM prevalence of 7% may soon translate to epidemic proportions and will have the capability to overwhelm the health system independently. The higher prevalence of T2D in older individuals is seen in both men and women and across racial and ethnic groups around the globe. India is labeled as the diabetic capital of the world, along with China, where the prevalence is more than 9.7%. The prevalence in Asian countries is attributed to swift lifestyle changes, automated work environments, poor urban planning, and academic pursuits. We observed that the tendency to develop T2DM among the elderly increases with increasing BMI. High body fat for equivalent BMI in Caucasians, excess abdominal adiposity, subcutaneous (deep subcutaneous), and intraabdominal fat among Indians at lower BMI levels have been significantly associated with marked insulin resistance [19]. Along with significant differences in body composition, the genetic susceptibility owing to low β-cell reserve and a predominance of β-cell dysfunction leads to insulin resistance and has been proven previously [20].

We observed that older people were more likelier to report diabetes and less likely to utilize health medication than their younger counterparts included in the analysis. In previous Chinese research, it was seen that the risk of diabetes attributable to metabolic risk factors (prediabetes and hypertension) decreased with age, and lifestyle risk factors increased substantially in adults aged 75 years and older, contributing to 29·7% of the population-attributable risk percentages for diabetes, with unhealthy sleep being a significant cause [3]. Then, a lower probability of medication utilization was observed in participants, not in a union.

The elderly from the ST caste were less likely to report diabetes but more likely to utilize health medication than SC older persons. On the contrary, OBC older persons were 0.08 and 0.05 times more likely to report diabetes and use health medication, respectively, than SC.

We observed a significant effect of **physical activities** in any form on the odds of developing T2DM among the elderly. Previous studies have depicted a significant negative association between the risk of developing T2DM and walking MET scores [21]. It is well known that Physical activity improves glycemic control and reduces the risk of cardiovascular disease (CVD) and mortality in patients with T2DM [21, 22]. Moderate to vigorous physical activity is recommended to manage T2DM; however, patients with T2D can be physically weak due to sarcopenia, or sarcopenic obesity, making it difficult to engage in the recommended levels of physical activity [23]. Therefore, the elderly should be motivated to increase their physical activity and decrease the duration of sedentary time in their routine. For frail elderly, even yoga has been seen to be beneficial through various Psycho-neuro-endocrine and immune mechanisms. Incorporating yoga practice in daily life helps attain glycaemic control and reduces the risk of complications in people with diabetes [24].

T2DM depicted socio-demographic disparities. The probability of developing T2DM was lower among females than males, but elderly females depicted better treatment utilization in our study. This is in contrast to the usual pattern seen in young age, who may rely on others to help them be compliant; in the latter case, women are less compliant in some situations. However, a woman’s adherence to traditional sex roles may be a hidden barrier to her compliance with a diabetic regimen. The woman may not be willing to change her family’s lifestyle to accommodate her health needs, may not feel that she has strong support from her family, or may be unwilling to discuss her illness with her husband [25]. Diabetes in women has more detrimental effects. Diabetes increases the risk of heart disease by about four times in women compared to two times in men, and women have worse outcomes after a heart attack. Women are also at higher risk of other diabetes-related complications such as blindness, kidney disease, and depression [26]. At the same time, the probability of reporting diabetes and using health medication was higher among more educated than non-educated participants. Education brings more awareness regarding diseases and their complications and the importance of drug adherence, as seen in previous studies from India and abroad [27, 28]. We observed a T2DM diagnosis and treatment **utilization gradient across the wealth quintiles.** In India, T2DM is mostly underdiagnosed and hugely affected by sociodemographic and health system factors. Socio-demographic inequalities are crucial in diagnosing and managing T2DM, especially in the elderly worldwide [29–31]. Socioeconomic status is attributable to three major health domains: healthcare utilization, environmental exposure, and health-related behavior. While diagnosis is related to healthcare utilization, adherence is related to health-related behavior. In our study, poor people have a lesser tendency to get diagnosed and managed for T2DM, which is a cause of concern. This can give us an idea about managing complications, including hospitalization during the disease that could not be assessed in the present study, and calls for deeper penetration of universal health coverage. Working participants were more likely to have diabetes, manifesting a negative association. Similarly, consuming smoking and alcohol consumption were also negatively associated with diabetes. Likewise, engagement in vigorous and moderate activities was associated with a higher probability of reporting diabetes. Due to the cross-sectional nature of the survey, it is difficult to establish the temporal association between these variables and diabetes. However, previous reports suggest that people change their behavior post-diagnosis and engage in healthier behavior due to better awareness of the disease and management strategies [32]. Our study depicted a lower prevalence of T2DM in current smokers and those frequently taking alcohol. It may be possible that such people have given up substance abuse post-diagnosis and have resorted to a healthier lifestyle. Otherwise, smoking and alcohol have proven detrimental effect on glycemic control and leads to metabolic syndrome [33].

**Although oral antidiabetic drugs (OADs) remain** the mainstay in the treatment of our study participants, insulin therapy was higher than previous estimates from India. But the quoted study was not restricted to the elderly [8]. According to the survey, 40% of patients with T2DM in India use insulin alone or in combination with OADs at any given time [34]. Within this context, it is pertinent to mention that Asians also tend to have varied efficacy responses to some diabetes medications, with more significant glycemic lowering effects with acarbose, glucagon-like peptide-1 (GLP-1) receptor agonists, and Dipeptidyl peptidase-IV (DPP-IV) inhibitors compared to other ethnicities [35, 36]. We observed that Insulin usage was slightly lower in the Muslim elderly. Among Muslims, fasting is part of their religious beliefs, which sometimes affects their insulin therapy decisions because of potential interference with their religious obligations. Muslim patients are also concerned about the origin of insulin and often believe it is a porcine derivative [37]. These concerns call for interventions with culturally tailored glycemic control and health education modules. Culturally and linguistically appropriate counseling, motivation to drug adherence, and cooking technique demonstrations have successfully improved glucose levels. It has also been suggested to have a higher threshold of HbA1c of 8% for older adults (age 75 years and older) and HbA1c of 7% for others to avert the most significant number of DALYs from insulin treatment by balancing the risk of hypoglycemia against the benefit of reducing microvascular complications [38].

**The present analysis’s particular strengths and limitations should be discussed.** The major strength of this study is the use of nationally representative datasets with reliable generalisability, as the data were collected following a robust and standardized methodology. Further, using Heckman’s selection model to address the non-random missing data makes the results more valuable. However, while this technique is an acceptable approach to assessing models for selection bias, it has significant limitations, including sensitivity to the model specification. For these reasons, we regard the selection model as an additional robustness check on our primary findings, not as definitive “proof” that selection bias is not still possible. Further, we cannot comment upon the effect of the non-availability of medicine on treatment utilization as this was beyond the scope of the existing datasets. We also cannot comment upon the impact of various other factors that may affect the risk of developing T2DM and their treatment-seeking behavior due to the limitations of the dataset. Secondary data analysis also limits us from making temporal associations between certain independent and dependent variables. There are many other factors like the availability of diagnostic services, cost of medication, out-of-pocket expenditure, operation issues for using insulin life refrigerator facilities, and health care workers’ capacity to diagnose and manage T2DM, including prescription inertia, that can affect treatment utilization but could not be assessed due to non-availability of variables in the available datasets.

**There are critical policy implications for this study**. Given the average earlier age of diabetes diagnosis in Indians, it is suggested that susceptible people should be targeted earlier in life with implementable and successful preventative strategies to reduce obesity and diabetes risk and limit diabetes-related complications in old age. There is clear evidence that intensive treatment of T2D early in the course of the disease has a substantial “legacy” effect in preventing long-term complications, even if glycemic control deteriorates over time [20, 39]. Policymakers should think about going beyond oral medications and help ensure easy access to insulin with minimum out-of-pocket expenditure. At the same time, researchers should think about developing better variants of insulin that can be used at room temperature to have a better reach in rural areas with less access to refrigeration facilities.

To conclude, our study depicts a high burden of T2DM in our study population. At the same time, treatment utilization was low and mainly limited to oral medications, with varied socio-demographic disparities. Considering the growing burden of the elderly population in a country like India, witnessing a significant demographic transition, we need to think about robust interventions to ensure treatment with adequate compliance and health advocacy for ensuring sustainable lifestyle modifications like physical activities and weight management that will help us to control the morbidity and mortality associated with T2DM and related complications.

## Data Availability

The data can be obtained from the **corresponding author** or can be accessed after filing a data request form available at the official website of the International Institute for Population Sciences-Mumbai.
